# Evaluation of the urban industrial coupling strategy based on the global production networks theory: A case study of the smart phone industry in the Guangdong-Hong Kong-Macao Greater Bay Area

**DOI:** 10.1371/journal.pone.0300588

**Published:** 2024-04-30

**Authors:** Sa Ma, Jinge Ding, Zhengdong Huang, Renzhong Guo

**Affiliations:** Research Institute for Smart Cities, School of Architecture and Urban Planning, Shenzhen University, Shenzhen, PR China; Guangzhou Institute of Geography, Guangdong Academy of Sciences, CHINA

## Abstract

In the context of uncertain economic environments urban agglomerations play a crucial role in economic development, reshaping industrial chains and fostering inter-city cooperation. This study employs the Global Production Network (GPN) theory to enhance our understanding of how cities integrate into regions, emphasizing the often-overlooked governmental influence in strategic coupling processes. In examining the evolution of China’s smartphone industry within the Guangdong-Hong Kong-Macao Greater Bay Area (GBA) this research categorizes 19,599 smartphone companies into five distinct groups. Through analyzing their spatial distribution and geographical linkage the study identifies four strategic coupling modes based on the localization of assets, considering spatial influence and technological complexity along horizontal and vertical dimensions. Structural and institutional elements within these modes are also explored. The research uncovers unique integration patterns among nine cities in the GBA’s mobile industry, revealing distinct spatial clusters rooted in technological, resource and innovation factors. Crucially, local policies play a pivotal role. Cities such as Shenzhen and Dongguan emerge as technology hubs, contrasting with Foshan and Zhongshan, which leverage resource advantages. The spatial impact, contingent on specific assets, underscores the necessity for nuanced top-down coupling methods in regional development. Moreover, the study emphasizes the significance of nurturing innovation links, not only between leading companies but also among midstream and downstream enterprises, enhancing cities’ strategic coupling capabilities.

## Introduction

The concept of “network economy,” introduced by Dicken et al. [1], has been a major area of study by scholars in recent years. The theoretical analysis framework of the global production network (GPN) has gained much attention, attracting such scholars as Huemer [2], Ketchen and Hult [3] and Ritter et al. [4], and has been developed by the “Manchester school” into a more complex theoretical framework based on actor network theory [5–8]. Compared to the research focus on regional social and institutional conditions in the “New Regionalism” of the 1990s [9], one of the main contributions of the GPN is its focus on the “relationship turn” [10] and it viewing interregional cooperation as a complex network composed of various actors that embedded, constructed and evolved together [11–13].

As a core concept of the GPN theory strategic coupling has established important variables that characterize the relationship between regions and the global production networks [14]. Coe et al. [5] emphasized that strategic coupling is the alignment of local advantages with the demands of leading firms in the global production network, resulting in the creation, enhancement and values capture of local areas. MacKinnon [9] introduced three coupling modes, namely structural coupling, strategic coupling and organic coupling. These three coupling modes progressively generate value by providing low-cost labour or resources to the global production network, engaging in strategic cooperation, and achieving autonomous innovation and value creation. Building upon this framework Yeung and Ceo [15] further proposed structural coupling (production platforms), functional coupling (international partnership relationships) and organic coupling (local innovation).

Based on this we highlight two gaps for further research. Firstly, in existing literature, the positive role of local enterprises in the development of strategic coupling has been underestimated [16]. The vast territory and dispersed regional economies enable local enterprises to construct their own value chains domestically [16]. Yeung [17] viewed strategic coupling as a dynamic process of urban/regional actors coordinating, mediating and arbitrating the strategic interests of local actors with their counterparts in the global economy. While this definition holds true for East Asian developing countries such as South Korea and Singapore, the process of strategic coupling in China appears to be different [18]. In the case of developing countries represented by China urban agglomerations and metropolitan areas have taken on more important economic development tasks, leading to an unprecedented transformation of the industrial chain and industrial cooperation between cities. For instance, twenty-four metropolitan areas, operating at the level of tens of millions, collectively occupy only 6.7% of the national land but house approximately 33% of the permanent population, contributing to around 54% of the GDP [19]. Furthermore, there is a notable trend of localization in the global production network. According to Zhang (2022) [20] the global value chain has entered the era of 3.0, characterized by localization and regionalization. This shift marks the end of the golden age of the global value chain, signifying a clear retreat in the process of globalization. Policies aimed at encouraging the re-shoring of industries, such as the United States’ 100-day reviews outlined in the executive order "Building resilient supply chains, revitalizing American manufacturing and fostering broad-based growth" and Japan’s allocation of substantial loans for “supply chain reform”, are expected to expedite the regional restructuring of the global production network and value chain. It is essential for enterprises of different cities, especially the backward cities in metropolitan areas, to participate in the different position of the industrial chain and encourage overall international competitiveness of the metropolitan areas. Secondly, according to the traditional GPN theory, multinational corporations form and drive GPNs. However, the role of governments is often overlooked in this process. It has been pointed out that the lack of coordination and communication among the different local government units hinders collaborative efforts by business actors for industrial development coordination, and that there exists an absence of local leadership to promote better coordination and communication among the different government units [21]. In their study of the Bekasi’s development Indraprahasta et al. [21] found that local governments had broader priorities and, therefore, could not ensure direct proactive promotion of industrial development through GPNs. In contrast, the Chinese government, through regional industrial synergy strategies, has proposed a series of regional collaborative development initiatives, encouraging integration of enterprises at the industrial chain level within regions, and promoting integration of lagging regions into the production network.

Thus, this research advocates that regions should go beyond the single concept of strategic coupling found in mainstream GPN literature (i.e. a region integrating into a production network led by a global leading firm) [22] and aims to address two key questions: (1) How do cities integrate into regions and leverage the GPN perspective on how regions “globalize” [5] based on regional endowments and influenced by local policymakers? (2) How can cities’ coupling capabilities be enhanced by promoting innovation links? The Guangdong-Hong Kong-Macao Great Bay Area (GBA) includes Hong Kong and Macao Special Administrative Region, Guangzhou, Shenzhen, Zhuhai, Foshan, Huizhou, Dongguan, Zhongshan, Jiangmen and Zhaoqing in the Guangdong Province and is an important carrier for China to build world-class urban agglomeration [23]. According to the "Joint Statistical Manual of the Guangdong-Hong Kong-Macao Greater Bay Area 2023" the economic aggregate of the GBA was almost 2 trillion USD in 2022. The GBA’s smartphone industry boasts a massive production scale and a complete industrial chain. It has some internationally advanced leading companies, such as Huawei, OPPO and VIVO, as well as core suppliers represented by ZTE, Konka and Skyworth, and many excellent local manufacturers and processing companies. According to the "2019 World Smart Mobile Terminal Industry Development White Paper" China’s smartphone shipments in 2018 reached 397 million units, with over 368 million units produced in the GBA, accounting for a quarter of the global smartphone shipments [24]. The development of China’s smartphone industry is one of the most successful cases in China’s modern manufacturing transformation. This research classifies the 19,599 smartphone companies into five categories (leading companies, core suppliers, specialized suppliers, general suppliers and contract manufacturers) based on their business scope and their position in the industrial supply chain. By analyzing the overall spatial layout of these five types of actors using the DO index and the coefficient of geographical linkage the study identifies four distinct strategic coupling modes among different cities basing on the localization of assets (spatial influence and technological complexity) along both horizontal and vertical dimensions. The research also discusses the role of structural and institutional elements within these modes. Furthermore, the study establishes innovation networks among actors and explores how innovation factors, as integral components of local assets, contribute to enhancing the value derived from participation in the production network of the GBA. This study also examines how nine cities within the GBA have integrated into urban cluster production networks and reveals how cities’ positioning within the GBA’s mobile industry chain is significantly influenced by local policymakers and varies across four quadrants, namely technological-territorial embeddedness, resource-territorial embeddedness, innovation explosion embeddedness and accidental embeddedness. Additionally, the research underscores that diverse actors in different cities contribute to spatial clustering and interconnections, offering distinct coupling paths, and emphasizes the significance of promoting innovation links, not only among leading companies but also between professional and general suppliers, to enhance cities’ strategic coupling capabilities.

## Theoretical background

The concept of the industrial chain originated from Marshall’s theory of the division of labour. Many scholars have discussed the industrial chain from the perspectives of the value chain and the supply chain [25, 26]. Based on the industrial chain scholars began to pay attention to value production and profit distribution in the production process [27–29]. In his book "Competitive Advantage" Porter [30, 31] introduced the concept of the "value chain" to explain the interrelationships among enterprises, suppliers, manufacturers, product distributors and consumers. Expanding on the theory of global commodity chains Gereffi [32] introduced the concept of the global value chain (GVC), which further explains the cross-regional distribution of vertical division activities in the current global production network [33]. Since the beginning of the 21st century the "Manchester School," represented by Peter Dicken, Jeffrey Henderson, Neil Coe and others, has been further highlighting the GPN on the basis of the concepts of the GVC and the global commodity chain (GCC) from a network theory perspective. The GPN is "a meso-level theory for analyzing the industrial-organizational dynamics at the levels of firms and their networks - respectively, actors and their organizational platforms - that underpin regional transformation" [34].

The introduction of the GPN was motivated by the fact that at the beginning of the 21st century many theories discussed only economic activities at the city or regional scale, especially in metropolitan areas [35]. They did not consider that industrial chains often need to go beyond the city, or even the regional, scale to reach a global scale. Additionally, actors in economic activities also have geographical, rather than administrative, boundary connections [35] and certain actors may be missing in urban clusters or regional areas [36]. Thus, the GPN focuses on the dynamic of organizations in the constantly changing process of globalization and their impact on regional development [11] and has been receiving increasing attention since then. However, in recent years the international economic environment has become increasingly uncertain and trade protectionism has become prevalent. As a result, global supply chains have experienced severe disruptions. This has given rise to a prevailing trend of anti-globalization and "inherent vulnerability and weak resilience" of the global production network [37]. Chaminade et al. [38] offer a critical perspective of GPN, arguing that the theoretical framework should not only include multinational actors, but also actors from developing economies. This highlights a limitation of the GPN, implying that the GPN theory lacks a specific explanation for developing countries, regions and other actors beyond leading enterprises. In developing countries, especially in the case of China, which is under increasing international scrutiny and competition, the regional development of urban agglomerations, such as the Yangtze River Delta area in China where "hundreds of millions of people live and work", should also be taken into account [39]. China has proposed a strategic deployment of industrial synergy, hoping to achieve regional coordinated development as a lever to achieve industrial closure, enhancing its overall international competitiveness by defining the division of labour among different cities, especially the backward cities which also hope to participate more in the industrial chain through the benefits of geographical proximity and their own resource benefits to improve their status in the value chain.

The GPN theory provides new views to explain regional industrial chain development through two key aspects. On one hand it serves as a valuable reference and logic to understand networks of firms and their partners for cooperation among regional actors [34]. It emphasizes the interconnections and relationships between different individuals and organizations and the collective outcomes of their associations within economic, social, and other systems [11, 40, 41]. In these actor networks various key actors are involved, including leading enterprises, strategic partners, professional suppliers, general suppliers, original equipment manufacturer platforms and consumers [11]. The GPN widely and emphatically applies the actor theory in the complex network constructed by geographical location and economic activities [42] and helps explain why actors behave in certain ways, how they influence each other and how they contribute to the overall functioning of the network, although the completeness of places may sometimes be “subordinated” when focusing on their role within “firms’ production networks” [43]. Regarding actors, the influence of lead firms on regional economies within actor relationships has been extensively examined [44], but, increasingly, existing research shows that the relationships of contract manufacturers in production networks are also complex and interdependent due to specialization in production [45].

On one hand, recently, researchers in EGG (evolutionary economic geography) have also been continuing their research on actors. MacKinnon et al. [46] expanded the coverage of coupling to the dynamic process that regional actors seek, use and match capital both in and out of the region during their research on path creation.

On the other hand, GPN studies focus on the strategic coupling among actors, especially within the value chain. The GPN theory examines value creation in enterprises and regions, as well as the embeddedness of actors, through three core elements, namely value, power, and embeddedness. Coe et al. [5] pointed out strategic coupling and explain the mechanisms of why some regions have more development opportunities than others, explaining the dynamic relationship between local and global firms, which significantly impacts regional development [13]. According to the GPN the strategic coupling between regions and GPNs is the reason for the region producing, promoting and maintaining regional values [47]. Sunley [48] criticized the early GPN methodologies for "lacking analysis models that prioritize considerations and determine causal mechanisms." In the concept of the GPN 2.0 greater attention is given to the dynamic impact of strategic coupling on the region, addressing problems such as a lack of problem orientation, unclear research boundary and difficulty in interaction quantification. Strategic coupling highlights how regions or clusters access development opportunities by integrating into the international industrial chain and providing regional resources (such as labour or assets) to lead firms [5, 9, 36, 49]. Yeung [50], after analyzing the case of the industrial transformation of East Asian countries, pointed out three dynamic modes of strategic coupling, namely “indigenous coupling in core regions premised on localized innovation and extra-regional linkages, functional coupling in emerging regions via inter-firm partnerships and extra-regional linkages, and structural coupling in peripheral regions through the provision of trans regional production platforms”. In addition, in the GPN’s analysis of strategic coupling two dimensions are discussed, namely the horizontal dimension and the vertical dimension. The vertical dimension emphasizes connections between actors (firms and organizations, etc.), while the horizontal dimension highlights territorial embeddedness based on geography [51]. The strategic coupling process involves cooperation, mediation and self-interestedness, and includes four dynamic strategies among actors, namely intra-firm coordination, inter-firm control, inter-term partnership and extra-firm bargaining. In recent years, regarding strategic coupling, Gong et al. [22] have suggested that "a region can simultaneously couple into multiple production networks" and actors can "combine different coupling scenarios."

Furthermore, Yeung’s research [50] delves into the specific role of the coupling theory in characteristic countries and regions during the industrial transformation process, emphasizing that strategic coupling encompasses not only enterprises but also dynamic networks and the relationship between innovation and regional coupling capabilities. Regarding the topic of innovation and the enhancement of strategic coupling capabilities Ernst [6] focuses on the innovation roles played by various actors within the network. Parrilli et al. [39] argue that lead firms tend to retain control over R&D networks and activities that affect their core capabilities, learning and global-scale innovation processes. Meanwhile, emerging firms catch up by engaging in R&D activities and increasing their innovation capabilities. From the EGG perspective innovation and knowledge are drivers of regional revolution and path development, leading to industrial upgrading and value capture trajectories [37, 52]. However, most studies predominantly focus on the role of lead firms within the GPN innovation network, exploring how they lead innovation. This approach tends to relegate other actors to passive roles, merely providing the elements that lead firms’ need (or actively contributing to couple themselves within the network). Such studies often overlook the impact of innovation by general suppliers and original equipment manufacturers on regional coupling into the GPN.

## Research methodology

### Data source and actor classification

The research data was obtained from the Wind database (https://www.wind.com.cn) and official enterprise business registration information based on the TianYanCha website (https://www.tianyancha.com). The actors in the mobile phone industry chain were classified based on the components and production links of the mobile phone industry. Grimes and Sun [53] divided the mobile phone supply chain into three levels, namely core supplier, non-core supplier and assembler. Liu et al. [14] corresponded the smart phone industry supply chain with different actors based on the estimated value of materials and separated the mobile phone industry into core suppliers of professional products, professional suppliers of general parts, general suppliers for multi-industries and suppliers for assembly and co-processing platforms, among others. This study added leading enterprises, whose main business scope is the sales of complete sets of private brand mobile phones, and determined the upstream and downstream actors of the industrial chain into five categories according to keywords (see Table 1). Finally, the research obtained 19,629 companies with clear business scope, capital, registered time, location and normal business operation status. To improve the geocoding accuracy irrelevant text in registered addresses was modified or removed and the structured address string was optimized. Based on the results of the geocoding companies with addresses that could not be accurately matched were removed, leaving 19,599 companies. After sample matching, selection, data cleaning and sorting the 19,599 smart phone companies were classified into 42 leading companies, 3053 core suppliers, 6568 specialized suppliers, 9583 general suppliers and 353 contract manufacturers. Geo-coding was used to transfer enterprises’ registered location to geographic longitude and latitude.

**Table 1:** Smart phone industry chain and actor classification.

Supplier Category	Primary Classification	Secondary Classification	Product Range
**Leading companies**	Brand Output	Brand Output	Sales of self-owned brand mobile phones
**Core suppliers**	Semiconductors	Application processors	APUs, GPUs, DRAM, operating systems, SoC chips
		Wireless communication chips	Baseband chips, transceiver modules, WLAN/BT/BB, GPS, Bluetooth, NFC chips, mixed signal chips
		Integrated circuits	Accelerometers, electronic gyroscopes, RF chips, analog semiconductors
		Discrete devices	MOSFETs, diodes, transistors, specialty devices
	Memory/Flash	Memory chips	Flash memory chips (NAND), dynamic storage chips (DRAM), flash, memory, SRAM
	Optical Components	Camera modules	Optical lenses, image processing chips, camera modules, camera motors, CMOS/CCD image sensors
	Display/Touch	Display screen	Backlight module, color filter, LCD panel, display panel, glass substrate, OLED, display screen chip
		Touch module	Touch chip, touch panel, fingerprint module
**Specialized suppliers**	Passive components	Integrated components	Connectors, SMT inductors, capacitors, resistors, magnetic components, filters, structural components
		RF components	Finite element method and other RF, high-performance RF connectors, antennas, power amplifiers
	Functional components	Precision components	Vibrators, linear motors, heat dissipation components, gyroscopes and accelerometers, electronic compasses, crystal oscillators
	PCB/FPC	Printed circuit board	Single-sided board, double-sided board, multi-layer board, HDI
	Acoustic Components	Electroacoustic devices	Receiver, Microphone, Speaker, Smart Speaker, Bluetooth Headset, Diaphragm
	Peripheral Electronics Components:	External electronic devices	Battery Module, Battery Cell, Power Converter, Charger, Data Cable, Power Adapter
**General suppliers**	External components	Structural components for appearance	Mobile Phone Shell, Accessories, Hinges, Pivot, Plastic Parts, Front and Rear Panel Brackets, Glass Back Cover
	Internal Parts	Internal Materials	Metal Materials, Plastics, Connectors, Conductive Isolation Bars, PVB Interlayer, Hollow Hinge
	Packaging/Printing	Packaging/Printing	Packaging Box, Manual, Warranty Card, Printing Materials/Equipment
**Contract manufacturers**	Contract Manufacturing	Contract Manufacturing	Chip Packaging and Testing, Chip Design, Packaging Solutions, Acoustic Component Manufacturing
		Assembly/Packaging	Component Assembly, Machine Assembly, Packaging Services

### Spatial agglomeration analysis

This study utilizes the DO index to analyze the spatial agglomeration among actors. The DO index relies on the random distribution of enterprise locations within the regional space to identify local spatial agglomeration patterns. Widely accepted in expressing spatial agglomeration it overcomes the limitations often found in traditional EG and MS indices, which are restricted by administrative unit divisions and a lack of results robustness. The measurement process in this research involves calculating the distance between enterprise locations based on their coordinates, utilizing k-density to measure distance density, employing Monte Carlo simulation to determine local confidence intervals, interpolating simulation results, establishing global confidence intervals and assessing industry agglomeration. For this study we converted the coordinate system to the UTM 49N projection coordinate system and calculated distance using the Euclidean distance between coordinate pairs (X, Y). Additionally, the coefficient of geographic association, which measures the spatial consistency between two variables, is calculated by counting the number of variables [54]. It can be expressed by the formula:

$$G=100-\frac{1}{2}\sum_{i}\left|S_{i}-P_{i}\right|=100-\frac{1}{2}\sum_{i}\left|(s_{i}/\sum_{i}s_{i})\times 100-(p_{i}/\sum_{i}p_{i})\times 100\right|$$ (1)

Here n is the number of cities, which is 9. Si represents the number of upstream actors in city i, and pi represents the number of downstream actors in city i.

### Strategic coupling modes

**Fig 1:**
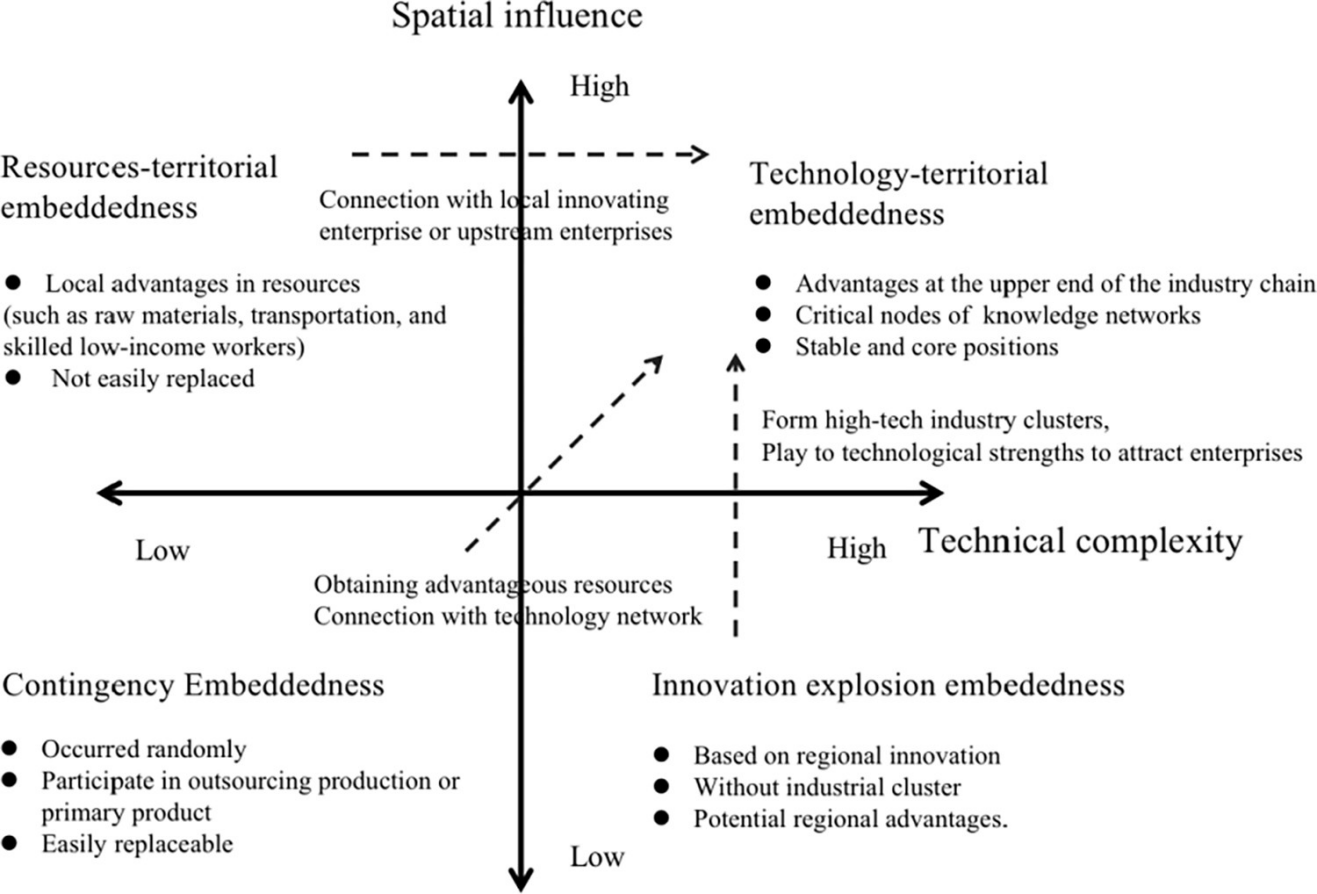
Four types of strategical coupling modes of cities.

This study asserts that cities can achieve strategic coupling in regional production networks through actors’ activities in both the vertical dimension (organization/actors) and the horizontal dimension (space) levels. Cities can enhance their value proposition in the production chain by taking more responsibility, not only upstream but also downstream. Four strategic coupling modes based on technical complexity and spatial influence are illustrated in Fig 1.

The first quadrant pertains to cities with a high level of technological competitiveness and spatial influence in a specific industry. These cities already possess advantages at the upper end of the industry chain, leading to the spatial aggregation of all facets of the industry chain. They play a pivotal role in regional industry development and upgrading, serving as the core of regional industry advancement. Other cities often need to establish networks with enterprises in these cities to achieve industry chain upgrading. Regions lacking such cities encounter challenges in breaking away from contract manufacturing or low-end production. Cities in this quadrant enjoy stable positions in the industry chain, making them difficult to replace.

In the second quadrant cities have low technological competitiveness but significant spatial influence, leading to the clustering of downstream enterprises in the industrial chain. This strategic coupling model primarily relies on local resources and can be termed the "resources-territorial embeddedness" coupling model. Typically, local advantages in resources, such as raw materials, transportation and a skilled yet low-income workforce, are abundant. Necessary skills can be acquired through imitation learning. Although this model is not easily replaceable because of the stability of unique local resources, these advantages may diminish with time and market changes, potentially impacting the stability and development of the industrial chain. In this coupling model bold ideas, innovative thinking, broad knowledge or entrepreneurial talent are required to achieve industrial innovation. This innovation can then be transferred to other local industries through knowledge networks or to connect with upstream enterprises in other regions to obtain regional industrial upgrading.

The third quadrant encompasses cities with low levels of both technological competitiveness and spatial influence in a specific industry. This industry lacks competitiveness or scale in the region and holds a lower position in the value chain. The development of this industry in the region can be considered sporadic. Cities following this coupling mode often participate in outsourcing production and primary product processing within the regional industry cluster, rendering them easily replaceable. For cities in this category there are two ways to increase their value in the industry chain: obtaining advantageous resources (such as tax incentives, institutional advantages and labour force recruitment) to establish a stronger spatial influence or improving their technological competitiveness through a technology network.

The fourth quadrant encompasses cities with high technological complexity but low spatial impact. This coupling mode is primarily grounded in regional innovation, often stemming from the fusion of industry, academia and research for technological innovation. It may also result from the relocation or establishment of branch offices of major leading enterprises. Such cities typically have a good innovation atmosphere conducive to innovation and possess potential regional advantages. This coupling mode is challenging to replace. To better leverage the technological advantages of these enterprises in the region it is necessary to increase the cooperation with other actors, form industrial clusters, especially high-tech and complex industries, and play to their technological strengths to attract enterprises in other links of the industrial chain, bolster, spatial impact and achieve urban industrial chain upgrading.

### Technical complexity

This study uses the Technical complexity index to measure the technical complexity within the mobile phone industry chain among five actors. Zander and Kogut [55], drawing on Winter’s [56] understanding of technological complexity, proposes that knowledge “is more complex when it draws upon distinct and multiple kinds of components.” Simon [57] suggests that different complexities are composed of parts that interact with varying degrees of difficulty. Most existing research focuses on economic complexity at the national level [58]. Hidalgo and Hausmann [59] innovatively introduced the Economic Complexity Index (ECI), which approximates the complexity of a country’s economic activities based on its production capabilities. They argue that products exported by a few, and most diverse, economies are considered more complex.

At the same time Ferrarini and Scaramozzino [60] and Stojkoski et al. [61] also demonstrate the importance of economic complexity for a country’s economic growth. Mewes and Broekel [58] further propose that a 10% increase in technological complexity is associated with a 0.45% increase in per capita GDP growth, considering that upper-level actors generally exhibit higher technical complexity than lower-level actors and the definitions provided above. The weight of the index $\frac{\left(x_{ij}/X_{i}\right)}{\sum_{i}\left(x_{ij}/X_{i}\right)}$ represents the explicit comparative advantage of city i concerning the technology of actor j. Therefore, it quantifies the technological complexity within the value chain involving these five actor types.

In this context i refers to the city, j refers to an actor and x_ij_ signifies the registered capital of actors j in city i, X_i_ represents the total registered capital of enterprises in city i, and Y_i_ reflects the per capita GDP of city i.


$$X_{i}=\sum_{j}x_{ij}$$ (2)

$${TC}_{j}=\sum_{i}\frac{\left(x_{ij}/X_{i}\right)}{\sum_{i}\left(x_{ij}/X_{i}\right)}∙Y_{i}$$ (3)

The technological complexity of the mobile phone industry in a certain city:

$${TC}_{i}=\sum_{j}\frac{x_{ij}}{X_{i}}∙{TC}_{j}$$ (4)

### Spatial influence

Spatial autocorrelation refers to the potential interdependence between the observed data of a variable in adjacent distribution areas. The Binary Local Moran Index and geographical connection rate are widely adopted to analyze the potential interdependence between the observed data of two variables (actors) in neighbouring distribution areas. The Moran’s I index ranges from 1 to -1, where *I*>0 indicates a positive correlation. A higher I value means more noticeable spatial clustering of the spatial variable with similar attributes has more obvious spatial attributes with similar characteristics. When I approach 0 this indicates no spatial correlation of the attributes. In addition, upstream enterprises have a stronger spatial absorption capacity for downstream enterprises, and downstream enterprises exhibit a higher degree of dependence on upstream enterprises in the industrial chain.

Therefore, urban industrial competitiveness can be measured by the spatial correlation among various types of actors. We assign weights to ten types of actor relations, considering the spatial influence of cities. These weight combinations include leading enterprise and leading enterprise (5x5), leading enterprise and core suppliers (5x4), leading enterprise and specialized suppliers (5x3), leading enterprise and general supplier (5x2), leading enterprise and contract manufacturers (5x1), core suppliers and core suppliers (4x4), core suppliers and specialized suppliers (4x3) and so on.


$${SI}_{i}=\sum_{j}\sum_{j^{\prime }}\sigma I_{ijj^{\prime }}$$ (5)

Here, i refers to the city, j and j’ refer to two actors and j can be equal to j’. *I_ijj_*′ is the Moran index, which ranges from -1 to 1, and *σ* is the weight coefficient.

### Upgrading the value based on the actor innovation network

**Fig 2:**
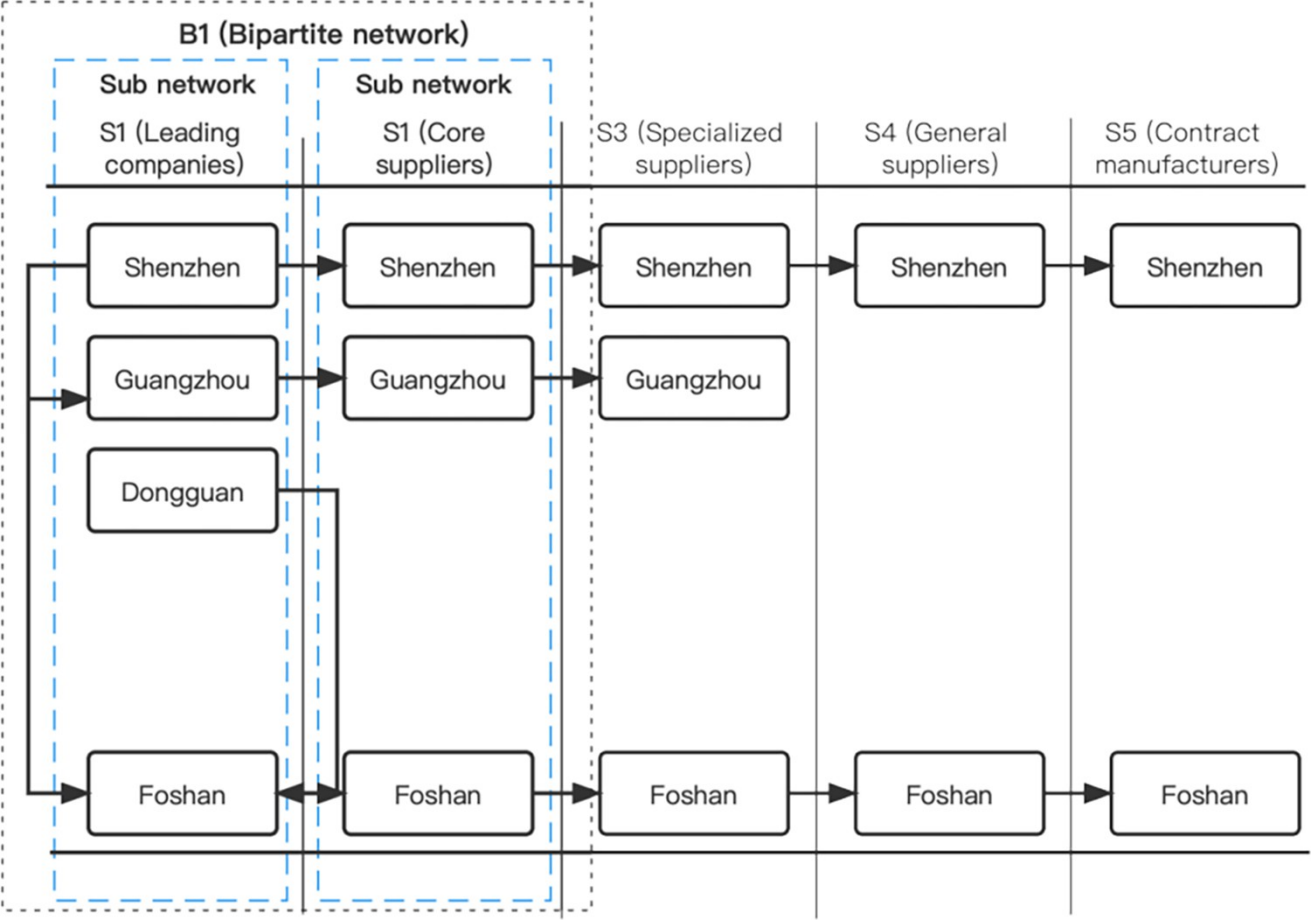
The schematic diagram of the innovation network topology model.

Enterprise patent data was extracted from the incoPat website (https://www.incopat.com) and categorized based on the top four IPC (International Patent Classification) codes associated with each patent. In total, 19,583 patents were collected. Companies that shared the same top four IPC codes were linked together as "company pairs." At both the actor and city levels we constructed an innovation network topology model for GBA using these "company pairs". This network model includes nodes representing different actors and cities, adjacent links that connected same actor across different cities, dependent links that connected different actors within the same city, sub-networks formed by connections between the same actor in different cities and bipartite networks formed by connections between different actors in the same cities (refer to Fig 2). Our network analysis involves the evaluation of network size and various network characteristics, such as density, local clustering coefficient, weighted centrality, and weighted structural holes.

## Results and discussion

### Spatial agglomeration of the smartphone industry

**Fig 3:**
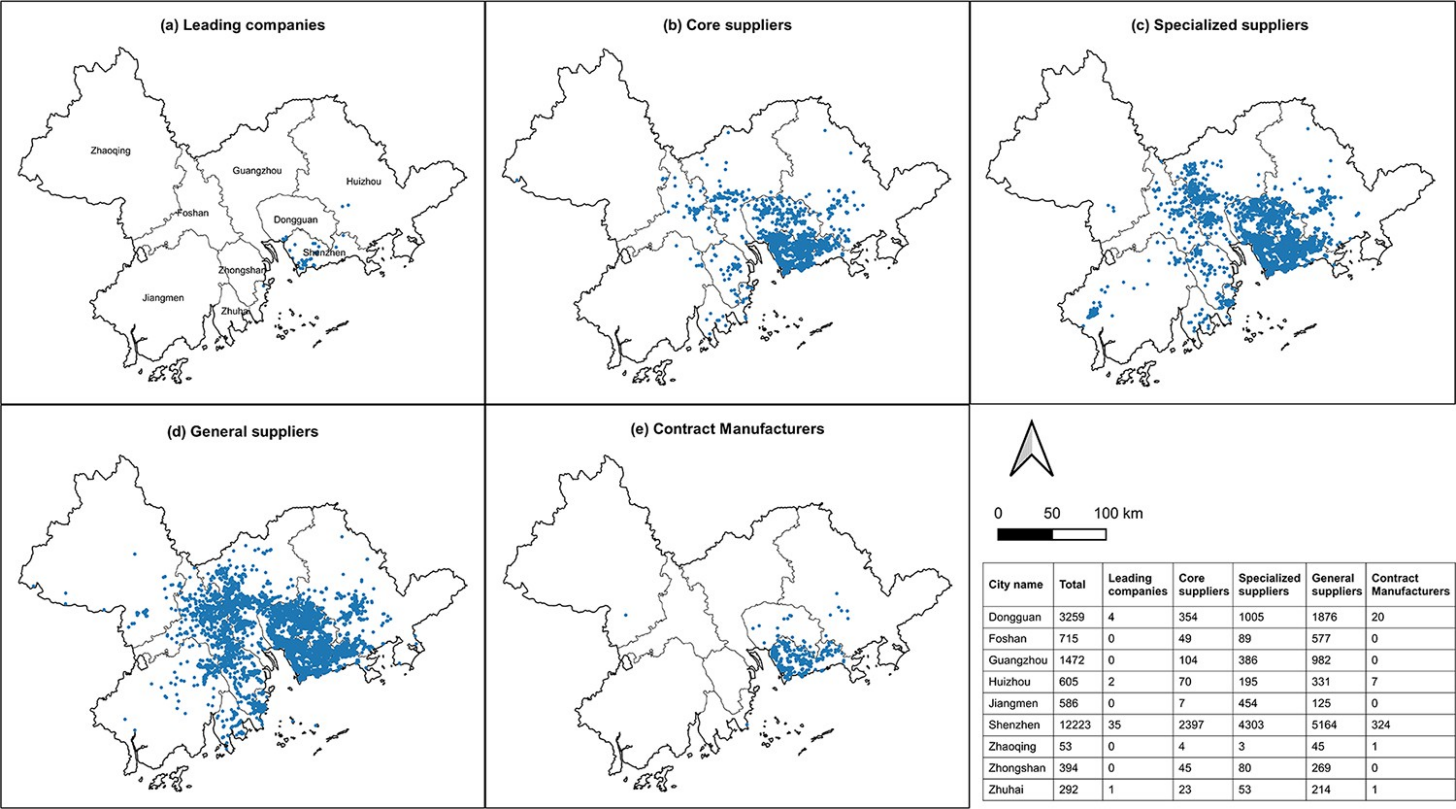
The overall spatial layout of five types of actors. Reprinted background map from the National Catalogue Service for Geographic Information (www.webmap.cn) under a CC BY license, with permission from the Ministry of Natural Resources of China, original copyright 2020.

The distribution of the five types of mobile phone industry actors in the GBA is shown in Fig 3. The majority of the GBA mobile phone industry is concentrated in Shenzhen (12,223), Dongguan (3,259) and Guangzhou (1472). Among them 42 leading companies are located mainly in Shenzhen (35), with some also in Dongguan (4), Huizhou (2) and Zhuhai (1). In addition to Dongguan and Shenzhen core production companies are also widely distributed in Guangzhou. The range of specialized suppliers has expanded to the south-western and north-eastern GBA, located in Jiangmen and Huizhou, with a significant increase in the distribution range. General component suppliers, such as Foshan (577), Zhongshan (269) and Zhuhai (214), are also major contributors to the GBA mobile phone industry. In particular Foshan has become a concentration area for general suppliers. Furthermore, packaging and contract manufacturing companies are mainly distributed in Shenzhen and Dongguan, being the two industrial clusters of the mobile phone industry. Economically underdeveloped Huizhou also has some distribution of these companies.

**Fig 4:**
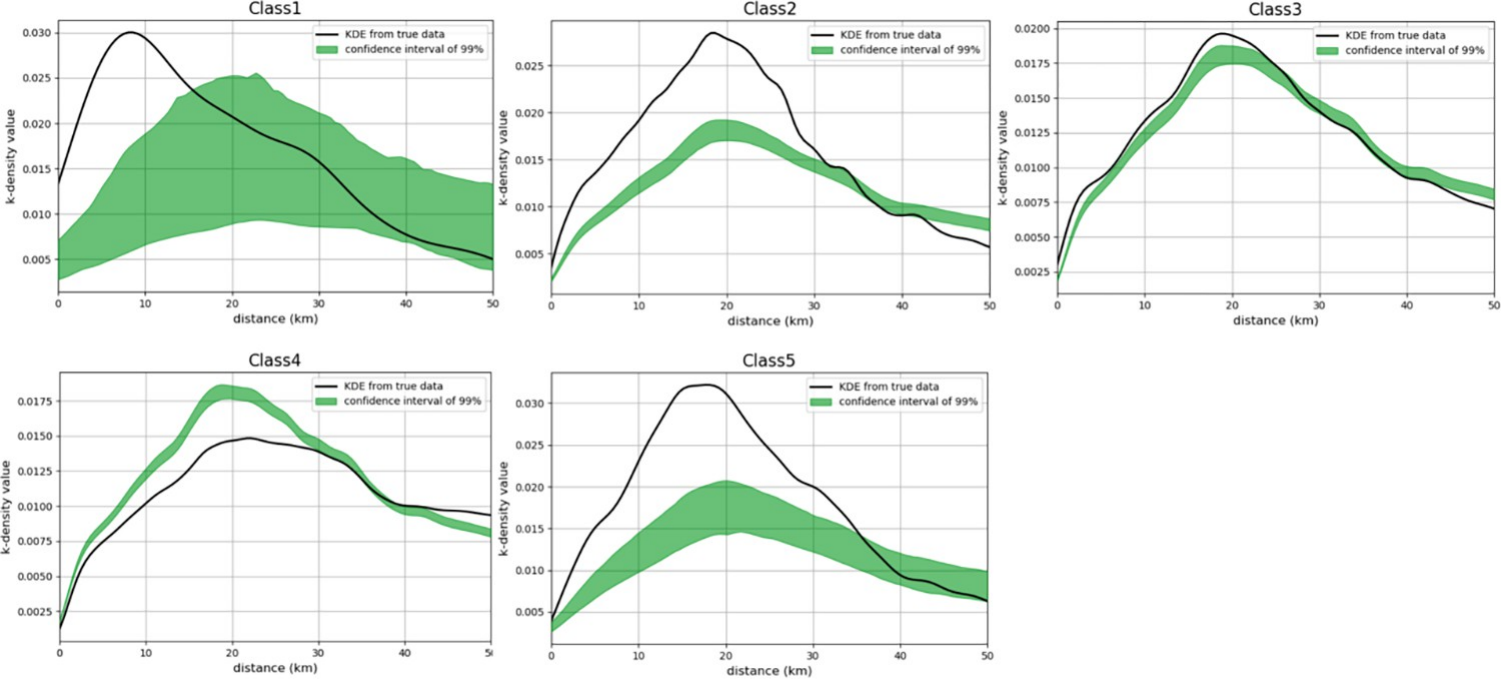
The DO index of five types of actors.

As shown in Fig 4, it is evident that various actors within the mobile phone industry have formed spatial agglomerations, reflecting the supplier clusters constituting the complete supply chain of the mobile phone industry in the GBA. On a global scale Zheng et al. [62] proposed that, apart from core regions in the GPN (Europe, the Americas, Asia), the influence of other regions in the GPN is primarily determined by geographical proximity. In our study of the GBA we observed that leading companies are clustered within a range of 0-14km, indicating that they tend to cluster in areas with higher geographic proximity. Core suppliers (0-30km), specialized suppliers (0-23km) and contract manufacturers (0-35km) are clustered within larger areas, exhibiting more dispersed spatial agglomerations. General suppliers are only clustered outside of the 40km range, indicating a lower demand for geographic proximity. This indicates that as businesses undertake lower-tier functions within the GPN their geographical dispersion increases.

The coefficient of geographic association between two actors is a measure reflecting the degree of proximity in regional configuration. Table 2 shows the coefficient of geographical linkage among actors in the mobile phone industry. Leading companies and contract manufacturers (91.27) exhibit the highest geographical connectivity, followed by leading companies and core suppliers (91.08), indicating a closer geographical relationship between leading companies, core suppliers and contract manufacturers. When local leading companies enter the production networks of related products based on their existing knowledge, their capabilities depend on strategic partners possessing the specific abilities necessary to meet the design, manufacturing, logistics and fulfilment requirements of leading companies’ customers [34]. Previous studies have shown that contract manufacturers often need to cooperate closer with leading companies for development and tend to establish themselves around core companies and core suppliers. Moreover, the role of core suppliers in the industry chain is increasing [63]. This research shows that, in addition to having close connections with leading companies in the GBA mobile phone industry, core component suppliers have high connectivity rates with professional suppliers (86.41) and contract manufacturers (86.58). However, general manufacturers have relatively low geographical connectivity rates with other industry actors. This is because general manufacturers often serve multiple industries, leading to a more independent geographical distribution as part of outsourcing strategies, as noted by Kostoska et al. [64], who emphasized that the rise of global production networks has actually reinstated a vast and unequal international division of labour, dividing the world into ‘headquarter’ and ‘factory’ economies. We found that in the GBA different types of actors exhibit concentrated leadership companies and core suppliers, whilst contract manufacturers are dispersed. Therefore, it is necessary to investigate how different forms of strategic coupling can enhance the participation of actors from different levels and scales, thus improving remote regions’ access to GPNs [18].

**Table 2:** The coefficient of geographical linkage of actors.

	Leading companies	Core suppliers	Specialized suppliers	General suppliers	Contract manufacturers
**Leading companies**	/	91.08	78.81	69.10	91.27
**Core suppliers**	/	/	86.41	75.37	86.58
**Specialized suppliers**	/	/	/	82.76	73.49
**General suppliers**	/	/	/	/	62.10
**Contract manufacturers**	/	/	/	/	/

### Strategic coupling modes of nine GBA cities

**Fig 5:**
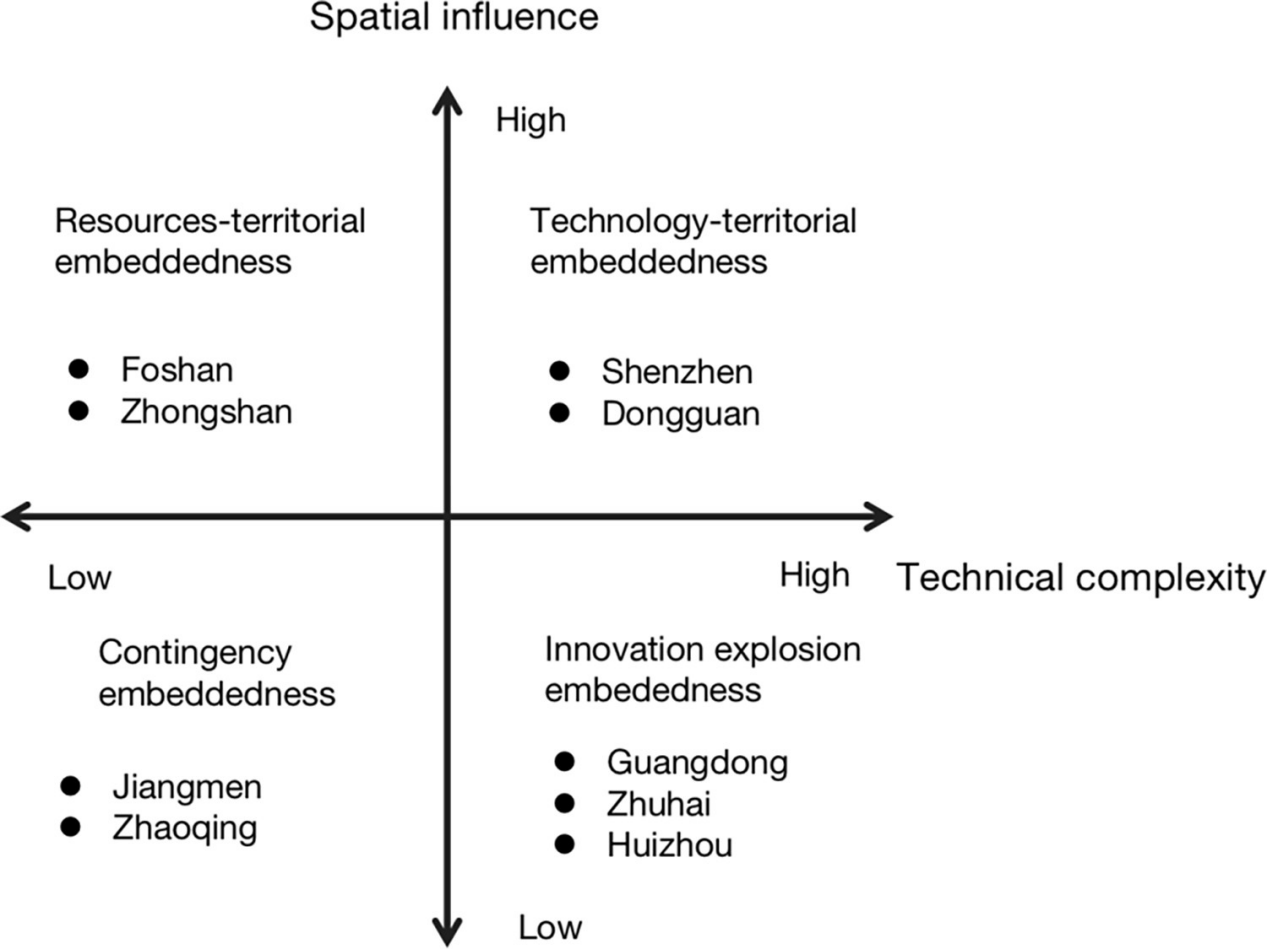
The coupling modes in the mobile phone industry among the nine cities in the GBA.

All global production networks are formed by cities, and all cities are integrated into global production networks [65]. However, different cities within the same region integrate into the industrial production networks in different ways. As mentioned by Xu et al. [51] in the case of strategic coupling modes in Jiangsu province, although both northern and southern Jiangsu are motivated by government “top-down” industrial cooperation strategies, they are currently at different strategic coupling levels. Northern Jiangsu is in the “structural coupling” stage, while southern Jiangsu is in the relatively mature “functional coupling” stage. This study reveals that the nine cities in the GBA are distributed across all four quadrants (Fig 5 and Table 3). In 2011 the first GBA “mobile phone industry integration implementation plan” (Implementation Plan for short) was issued by the Economic and Information Commission outlining the goal of “accelerating the promotion of integrated development of the mobile phone industry and enhancing the value chain of the mobile phone industry”. By 2015 the mobile phone industry in the GBA was aiming to establish 1-2 leading enterprises. This policy framework supported Shenzhen’s role as a hub for mobile phone resources, making it the design centre for the mobile phone industry, with final assembly taking place in Dongguan. This approach fostered local technological advantages and spatial attractiveness (Implementation Plan, 2011), resulting in the mobile phone industries in Shenzhen and Dongguan demonstrating strong performance in both technical complexity and spatial influence, positioning them in the first quadrant characterized by technology-territorial embeddedness. The success of China’s mobile phone industry largely depends on the rise of leading enterprises. Whilst Xu et al. [51] emphasizes that regional economic growth should be primarily driven by endogenous factors rather than external forces, regional policymakers have played a crucial role in facilitating the integration of Shenzhen and Dongguan into the GBA smartphone production networks.

**Table 3:** Technical complexity and spatial influence of nine cities.

Cities	Technical complexity	Spatial influence	Type of strategical coupling modes
**Guangzhou**	11.531	0.117	Innovation explosion embeddedness
**Shenzhen**	12.851	0.8117	Technology-territorial embeddedness
**Zhuhai**	11.548	0.002	Innovation explosion embeddedness
**Foshan**	10.904	0.394	Resources-territorial embeddedness
**Huizhou**	11.373	0.019	Innovation explosion embeddedness
**Dongguan**	11.153	0.914	Technology-territorial embeddedness
**Zhongshan**	10.852	1.283	Resources-territorial embeddedness
**Jiangmen**	10.449	0.092	Contingency embeddedness
**Zhaoqing**	10.696	-0.844	Contingency embeddedness
**Average**	11.151	0.336	

The regional assets of second-tier cities can be considered as driving factors for attracting investments [66] and are also the development factors adopted by Foshan and Zhongshan in the mobile phone industry. Both of these cities exhibit above-average spatial influence but relatively weak technological competitiveness, particularly Zhongshan, which aligns with the concentration of mobile phone companies, especially downstream enterprises. These cities are integrated into the industry chain through the resources-territorial embeddedness mode. Foshan, being close to Guangdong, enjoys convenient transportation, fostering the development of private capital and trade, leading to the clustering of small and medium-sized enterprises. The government introduced a geographically diverse mobile phone industry cluster making Foshan and Zhongshan significant manufacturers (Implementation Plan, 2011). The region’s performance in the mobile phone industry further underscores this phenomenon. However, although Zhongshan boasts a strong manufacturing base, primarily in intensive industries, it has not reaped the benefits of technology spill over from high-tech enterprises in Guangzhou and Shenzhen, limiting its technology transfer capabilities. From this perspective, localized and regional assets, such as transportation and policies supporting, serve as the foundational basis for territorial development, shaping regional production networks [21].

Three cities, namely Guangzhou, Zhuhai, and Huizhou, are part of the innovation explosion embeddedness category. The government has proposed establishing mobile phone industry production bases in Huizhou and Zhongshan within industrial parks, forming strategic enterprises. The aim is to address issues relative to the smartphone industry at various government levels through intergovernmental consultations. Through analyzing the indicators of spatial influence and technical complexity we found that these three regions have established innovation and knowledge centres. Among them, Guangzhou has a high economic development level, whilst Zhuhai and Huizhou have average or lower economic development levels. Although Guangzhou has a significant advantage in innovation and technology, being a mega-city, it should further enhance its spatial influence. It is worth noting that the government proposed supporting front-end design and development in Guangzhou and Shenzhen. However, although Guangzhou has established itself as an innovation hub, when compared with Shenzhen it has not created significant spatial influence within the mobile phone industry. This is partly due to the lack of leading enterprises and a strategic absence of core suppliers and specialized suppliers, as well as an absence of nearby manufacturers and complementary businesses. Huizhou and Zhuhai, on one hand, should rely on leading enterprises to leverage their advantages in technical complexity, forming a trend of industry agglomeration, and driving regional economic development. Thanks to government policies relatively economically underdeveloped cities, such as Zhuhai and Huizhou, have become leading suppliers of components, such as mobile screens and batteries (half of the world’s mobile glass screens come from Huizhou [67]). However, due to their supplier-oriented approach (dominated by leading enterprises), these cities’ spatial influence within the urban cluster remains limited. The development of government-driven knowledge centres in spatial terms will take time, posing a challenge that the top-down coupling approach needs to consider.

Jiangmen and Zhaoqing fall into the third quadrant. Referring to Fig 3, these two cities exhibit a less widespread presence of industry actors, with both cities having below-average levels of spatial influence and technological innovation. In the upstream segment of the industry chain Jiangmen has only a few core enterprises and there are not many industry clusters in downstream, indicating a random appearance of enterprises in these two cities, representing a contingency embeddedness. The degree of coupling can range from complete to limited or absent, encompassing traditional material connections and less obvious "non-traded interdependencies" [68]. Although structural factors are crucial, they are not the sole reason why companies choose specific locations for investment. Local institutions play a significant role in attracting and connecting the industry with regional assets [66]. The significance of studying these regions lies in enhancing certain capabilities to better integrate into the region, even though these efforts are currently limited.

### The role of the innovation network

Leading East Asian economies are shifting their priorities from being part of Global Production Networks (GPNs) to becoming part of Global Innovation Networks (GINs) [69]. An innovation network is a collaborative system of information exchange and mutual cooperation among actors [70]. Emphasis is placed on enhancing the innovation capabilities of the entire network through communication and collaboration among internal entities. McEvily categorizes innovation networks as organizational networks, stating that businesses exchange information within these networks to enhance their innovation efficiency [71]. Many studies have shown that there exists a close relationship between a region’s innovation capability and its position and embeddability within the industrial production network [72]. Improving its innovation capability can enhance a region’s coupling capability and change the path dependence of a region’s economic development, thereby changing the city’s position in the global production network. However, only a few studies have focused on the different roles played by innovation networks composed of different actors in regional coupling.

**Fig 6:**
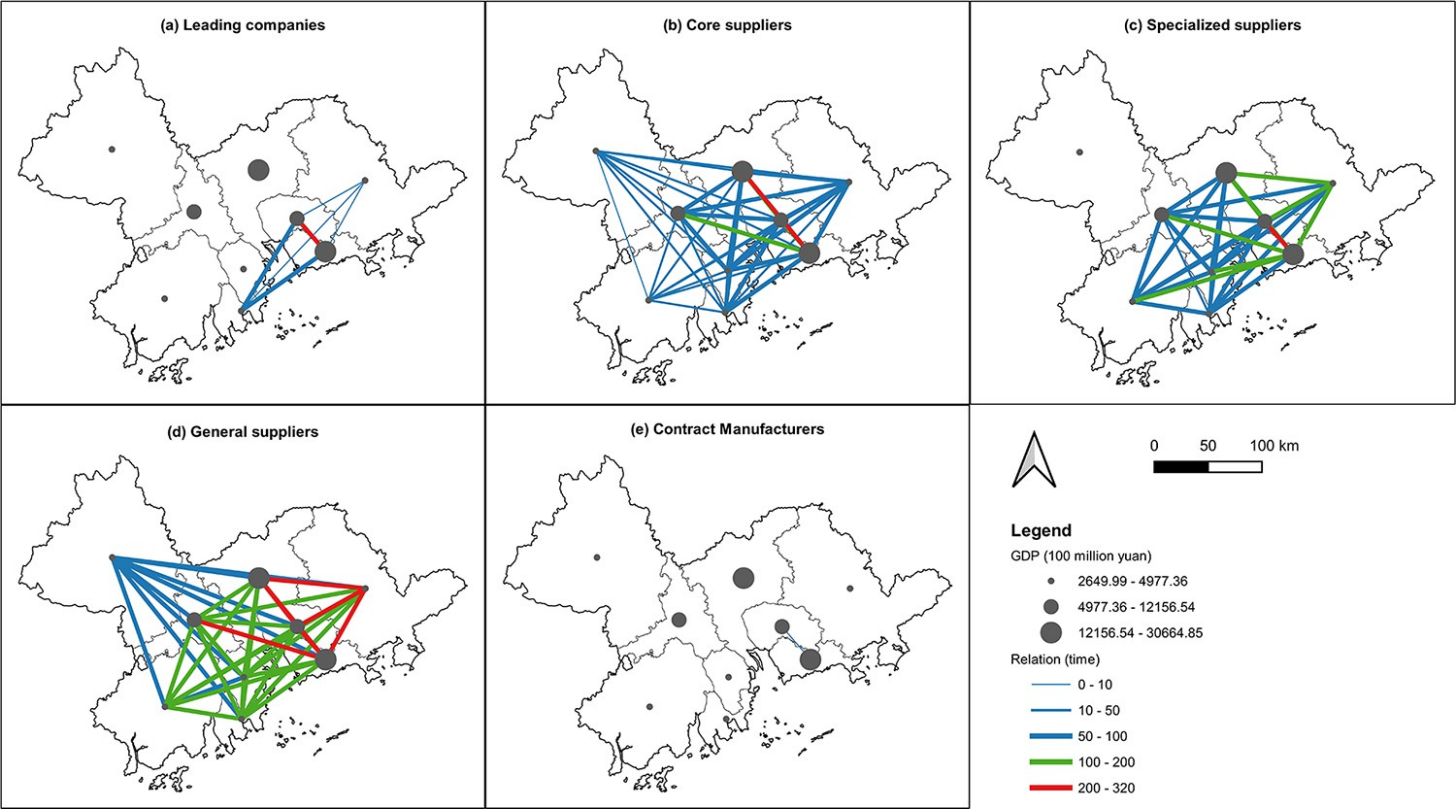
Innovation network topology model among cities. Reprinted background map from the National Catalogue Service for Geographic Information (www.webmap.cn) under a CC BY license, with permission from the Ministry of Natural Resources of China, original copyright 2020.

The overall network consists of 45 nodes and 495 lines (Fig 6 and Table 4). The network’s density is 0.5, indicating that around half of the possible connections between nodes are present. Shenzhen and Dongguan have the highest weighted centrality in the overall network and play important roles in the knowledge networks of the five types of actors, ensuring their coupling into the industrial chain through technology-territorial embeddedness. This is especially true in the innovation network constructed among leading companies and core suppliers. Guangzhou, Huizhou and Zhuhai also perform well in terms of their innovation networks, with their overall network degree centrality reaching 0.636, 0.544 and 0.487 (Table 5), respectively. This allows them to be coupled into the industrial chain through innovation explosion embeddedness. However, Guangzhou, along with Shenzhen and Dongguan, has become the core of network contacts, leading to a large weighted structural hole (3.117) and a lower stability and information transmission capacity of the network.

**Table 4:** The analysis of network characteristics.

Network	Size		Overall network characteristics				weighted structural holes
	nodes	lines	density	local clustering coefficient	weighted centrality	average path length	
**N**	45	495	0.5	0.71	0.19	1.002	11.059
**S1**	9	6	0.167	0.444	0.157	1	1.249
**S2**	9	36	1	1	0.225	1	3.117
**S3**	9	28	0.778	0.889	0.279	1	2.864
**S4**	9	36	1	1	0.228	1	2.628
**S5**	9	1	0.028	0	0.125	1	1
**B1**	18	36	0.235	0	0.169	1.538	6.5
**B2**	18	72	0.471	0	0.125	1.471	8.5
**B3**	18	72	0.471	0	0.196	1.471	8.5
**B4**	18	17	0.111	0	0.35	1.709	5.235

**Table 5:** Weighted centrality of different cities.

	Overall network	S1	S2	S3	S4	S5	B1	B2	B3	B4
**Shenzhen**	1.343	0.171	0.397	0.520	0.656	0.125	0.327	0.394	0.538	0.412
**Dongguan**	0.912	0.171	0.367	0.441	0.604	0.125	0.248	0.261	0.416	0.069
**Guangzhou**	0.636	0.087	0.307	0.396	0.584	0.000	0.102	0.265	0.312	0.045
**Huizhou**	0.544	0.009	0.219	0.336	0.529	0.000	0.051	0.193	0.350	0.047
**Zhuhai**	0.487	0.000	0.215	0.296	0.490	0.000	0.116	0.116	0.235	0.040
**Foshan**	0.447	0.000	0.197	0.266	0.451	0.000	0.046	0.160	0.273	0.039
**Zhongshan**	0.401	0.000	0.160	0.255	0.390	0.000	0.041	0.154	0.251	0.033
**Jiangmen**	0.303	0.000	0.071	0.222	0.340	0.000	0.013	0.102	0.223	0.025
**Zhaoqing**	0.130	0.000	0.058	0.000	0.260	0.000	0.010	0.019	0.073	0.016

Although innovation by leading and core companies has a stronger coupling effect on cities, Huizhou and Zhuhai play an important role in the knowledge networks of specialized and general suppliers. For example, in the innovation network of general suppliers the degree centralities of Huizhou and Zhuhai are much higher than other cities’, at 0.529 and 0.490, respectively. This also couples economically underdeveloped Huizhou and Zhuhai into the mobile phone industry chain of GBA in the form of "innovation explosion embeddedness." This also reflects that, although industrial innovation can promote strategic coupling, it is not only the innovation of core or leading companies in the upstream of the industrial chain but also the innovation links between companies in the middle and downstream of the industry. Specialized or general companies usually serve multiple industries, which means that innovation links between them can allow a region to have a presence in many industry chains. Therefore, while the government has traditionally prioritized innovation by core and leading companies, it should not overlook the role of general and specialized companies in regional development.

Foshan and Zhongshan, as areas where multiple types of actors gather, play a more important role in the innovation network between core companies and specialized suppliers, as well as between specialized and general parts suppliers. However, for increasing technological complexity, the innovation network between single types of actors may have a greater impact than cross-actor connections. Jiangmen and Zhaoqing do not get involved in the leading companies’ network and have relatively weak connections (almost less than 50 times) with other cities in terms of core suppliers. Contract manufacturing companies have almost no technological innovation, hence they do not constitute a complex network, and the network density is only 0.028. Only Shenzhen and Dongguan have a few connections.

### Conclusions

The research reveals diverse integration patterns of the nine cities within the GBA into urban cluster production networks, distributed across four quadrants: technological-territorial embeddedness, resource-territorial embeddedness, innovation explosion embeddedness and contingency embeddedness. Local policymakers significantly influence the positioning of cities within the GBA mobile industry chain, as evidenced by the "mobile phone industry integration implementation plan" (2011). This plan has led to notable differences in technological complexity and spatial impact within the GBA’s mobile industry, with Shenzhen and Dongguan serving as hubs for technological-territorial embeddedness and Foshan and Zhongshan falling under resource-territorial embeddedness. Furthermore, Guangzhou, Zhuhai and Zhongshan are positioned within the innovation explosion embeddedness quadrant.

However, regional performance is contingent upon specific assets, such as transportation and policy support. Despite being an innovation hub, Guangzhou lacks strong spatial impact in the mobile industry, indicating strategic gaps. This study uncovers varied actors across different cities, providing distinct coupling paths, with leading companies clustering while general suppliers are relatively dispersed. Strong spatial connections exist between leading companies, strategic partners and core suppliers. General manufacturers exhibit geographical independence, emphasizing diverse roles’ dispersion in geographical space.

In contrast to past research that focused on innovation links between upstream leading companies and core enterprises, this study suggests that enhancing innovation links between professional suppliers and general suppliers can also strengthen cities’ strategic coupling capabilities. Therefore, when formulating regional development strategies governments should not only focus on innovation among upstream companies but also promote innovation among midstream and downstream enterprises in the industry chain.

The theoretical contributions of this paper include recognizing that the conventional strategic coupling perspective alone cannot fully explain regional economic adaptation in uncertain periods [22], and blindly enhancing regional adaptability to global production networks might hinder effective strategic connections between regional economies, disrupting regional developmental potentials [51]. This study theoretically constructs four models, namely technology-territorial embedding, resource-territorial embedding, innovation explosion embedding, and contingency embedding. These models provide a theoretical foundation for the coupling and coordinated development of cities in uncertain urban cluster construction processes. Additionally, this paper emphasizes the impact of government on strategic coupling. Yeung [13] defines national-level coupling as the dynamic process by which national firms partially or completely disengage from their domestic political-economic structures over time and then recombine with lead firms in global production networks [18]. However, the situation in the GBA differs. This study underscores the significant role of government in facilitating the formation of leading enterprises and industrial layouts, emphasizing the importance of regional policies in strategic coupling [62].

On a practical level cities must adapt according to the demands of the new era, particularly in the wake of the significant lifestyle shifts caused by the emergence of Covid-19 [73, 74]. This study provides insights into the smartphone industry’s success in the GBA, offering methods for cities to integrate into urban clusters and paradigms for regional collaborative development. City governments have had to change their service delivery methods, especially under lockdown restrictions, implementing strategies for regional collaborative development [23]. Furthermore, the findings offer insights into developing dynamic regional industrial policies in alignment with the characteristics of strategic coupling stages. The success of the smartphone industry in recent years has resulted from strategic coupling and collaboration among cities at various stages of the industry’s value chain. Regions have integrated into production networks in both technological and spatial levels. These findings offer insights into developing dynamic regional industrial policies in alignment with the characteristics of strategic coupling stages [51].

Whilst this study does serve as a valuable resource for regional policymakers, its limitations should be acknowledged. The key value of products in the mobile industry largely resides in the software, systems and services offered on smartphones, as well as the innovative applications [75]. This study did not incorporate these aspects. Moreover, industries beyond manufacturing were overlooked. Second, the emphasis on micro-scale processes of the GPN might overlook macro-scale patterns that are evident at larger scales [62]. Efforts by various cities in the GBA to capture additional value across multiple value chains by undertaking all production stages locally might reduce the opportunities for regional participation in global production networks, being a phenomenon observed in other regions such as Indonesia (mentioned by Patunru and Rahardja [76]).

Future research on this topic could explore various dimensions, including an investigation into whether global production networks are localizing and how cities can capitalize on these evolving dynamics. A crucial aspect of this investigation could involve conducting dynamic analyses over time to gain an understanding of how to enhance the coupling capabilities of different cities within a region. Furthermore, delving deeper into the repercussions of changes and reconfigurations in industrial chains on regional economies could also be a valuable topic for future investigation.
