# Expression of Concern: Evaluation of the urban industrial coupling strategy based on the global production networks theory: A case study of the smart phone industry in the Guangdong-Hong Kong-Macao Greater Bay Area

**DOI:** 10.1371/journal.pone.0352126

**Published:** 2026-06-25

**Authors:** The PLOS One Editors

The *PLOS One* Editors issue this Expression of Concern for this article [1] because of concerns identified about the peer review process. Readers are advised to interpret the article [1] with caution.
